# Biofilm-related characteristics of *Candida parapsilosis* in postoperative ocular infections

**DOI:** 10.3389/fcimb.2026.1753328

**Published:** 2026-02-02

**Authors:** Yuxuan Wu, Min Kang, Zhiqun Wang, Yang Zhang, Kexin Chen, Qingfeng Liang, Xinxin Lu

**Affiliations:** 1 Peking University First Hospital, Beijing, China; 2 Beijing Tongren Hospital, Capital Medical University, Beijing, China

**Keywords:** 1,3-β-D-glucan, biofilm, Candida keratitis, Candida parapsilosis, postoperative ocular infection

## Abstract

**Objective:**

The research aims to elucidate the pathogenic mechanisms of *Candida parapsilosis* infection after keratoplasty and provide evidence-based guidance for the clinical management of *Candida* infections in ophthalmic practice.

**Method:**

Biofilms were cultured from 45 strains of *Candida*. The total biomass of the biofilms was measured using the crystal violet staining method, and the biofilm activity was assessed via the XTT reduction assay. Cell surface hydrophobicity and adhesion were evaluated for all *Candida* strains. The minimum inhibitory concentration (MIC) of planktonic *Candida* was determined using the colorimetric microbroth dilution method, while the MIC of biofilm-embedded *Candida* was measured via the XTT reduction assay. The release of 1, 3-β-D-glucan was detected using the G-test, and the chemotactic ability of 1, 3-β-D-glucan on neutrophils was evaluated via the Transwell assay. Molecular typing of *Candida parapsilosis* was performed using microsatellite genotyping. Statistical analysis was conducted using the Kruskal-Wallis (K-W) test.

**Results:**

In 45 postoperative ocular *Candida* isolates, *Candida parapsilosis* accounted for 48.9% (22/45), *Candida albicans* 35.6% (16/45), *Candida tropicalis* 11.1% (5/45), and *Candida glabrata* 4.4% (2/45). The total biofilm biomass and metabolic activity of *Candida parapsilosis* at 4°C were significantly higher than those of the other *Candida* species. In the cell surface hydrophobicity assay, *Candida parapsilosis* was more hydrophobic than *Candida albicans* and *Candida glabrata*, but less hydrophobic than *Candida tropicalis*. Among *Candida parapsilosis* isolates, 77.3% (17/22) showed strong adhesion ability and 81.8% (18/22) showed strong biofilm-forming ability (OD450>0.16). *Candida* colony and spore morphology were found to correlate with biofilm-forming ability. Strains with strong biofilm-forming ability had wrinkled, dry colonies; Gram-stained spores appeared as pseudohyphae; and lactophenol cotton blue staining showed spores that were uniformly and deeply stained. In the biofilm-antigenicity analysis, the non-biofilm-forming group’s 1, 3-β-D-glucan release was significantly higher than that of the strong biofilm group, thereby attracting more neutrophils. In antifungal susceptibility tests, except for *C. tropicalis*, biofilm-grown *Candida* showed higher minimum inhibitory concentrations (MICs) than planktonic cells for all antifungal drugs. Caspofungin was active against all isolates in both states.

**Conclusions:**

This study demonstrates that *C. parapsilosis* has greater adhesion ability and a stronger capacity to form biofilms at 4°C (with higher metabolic activity) than other *Candida* species. When laboratory findings reveal a *Candida* isolate with a rough colony morphology, its biofilm-forming ability should be tested and antifungal susceptibility should be assessed under biofilm-growing conditions rather than in planktonic culture.Clinically, we recommend shifting antifungal therapy to caspofungin for such infections.

## 1 Introduction

In recent years, the incidence of *Candida* keratitis has been rising (Fontana et al., 2019; Song et al., 2021; Masoumi et al., 2024). Fernanda M. Bezerra et al. reported that 84.6% of patients with *Candida* keratitis had a history of ocular surgery, and 76.9% of those patients had *C. parapsilosis* isolated (Bezerra et al., 2023). Tarika Thareja et al. proposed that postoperative corneal *Candida* infections are due to donor corneas being contaminated during storage; donor corneas are typically stored at 4°Cin Optisol-GS preservation solution for 10–14 days (Lau et al., 2019; Thareja et al., 2020). Branchini, Pfaller et al. found that *C. parapsilosis* can proliferate in storage media and adhere to prosthetic materials to form biofilms, with the extracellular polymeric matrix helping it to firmly attach (Deogaonkar and Roy, 2023; Asogan et al., 2024). After local colonization, *C. parapsilosis* can further recruit planktonic cells and mature the biofilm, providing a favorable three-dimensional structure for persistent survival. However, due to the small number of post-corneal transplant *Candida* infection cases, laboratory data on biofilms in this context are very limited, and the role of biofilm in postoperative *C. parapsilosis* infections remains inconclusive.

Beijing Tongren Hospital of Capital Medical University, the largest ophthalmic center in Northern China, maintains a comprehensive strain collection. In this study, we collected 45 *Candida* strains from postoperative ocular infections over a 15-year period. Through assays of adhesion, biofilm-forming ability, antigenicity, antifungal susceptibility, and microsatellite typing, we investigated possible factors contributing to *C. parapsilosis* infections following ophthalmic surgery. The study aims to elucidate the pathogenesis of *Candida parapsilosis* infection after corneal transplantation and guide the clinical rationality of antifungal therapy.

## 2 Materials and methods

### 2.1 Strain source

We collected 45 *Candida* isolates from postoperative ocular infections between January 2010 and December 2024, including 22 C*. parapsilosis*, 16 C*. albicans*, 5 C. *tropicalis*, and 2 C*. glabrata*. *C. parapsilosis* ATCC 22019 was used as a reference control strain. It is used as a quality control strain for mass spectrometry identification and biofilm formation assays. In this study, all *C. parapsilosis* isolates were identified to the species level, not merely as part of the complex. Each isolate was identified by matrix-assisted laser desorption/ionization time-of-flight mass spectrometry (MALDI-TOF MS). A small amount of colony material from a fresh culture was picked with a sterile swab and smeared evenly onto a MALDI-TOF target plate, then air-dried at room temperature. After drying, 1μL of 70% formic acid was added to the spot, followed by 1μL of matrix solution (saturated α-cyano-4-hydroxycinnamic acid in 50% acetonitrile + 47.5% ultrapure water + 2.5% trifluoroacetic acid). After the sample dried again, identification was performed using a Smart MS 5020 automated microbial MALDI-TOF MS system (Zhuhai DL Biotech). A score≥2.0 was considered a reliable species-level identification (Xie et al., 2019; Rodríguez-Temporal et al., 2023).

### 2.2 Biofilm assay

Each *Candida* isolate was inoculated on Sabouraud dextrose agar and grown at 35°C for 48 h, then washed twice with sterile phosphate-buffered saline (PBS) (10, 000 g, 5 min). The cells were resuspended in Sabouraud dextrose broth and adjusted to 2×10^6^ cells/mL. Then, 100μL of this suspension was added to each well of a sterile 96-well plate (three replicate wells per strain) and incubated at 37°C for 24 h. After incubation, the medium was discarded and the wells were gently washed three times with PBS. Biofilm formation was quantified by measuring the absorbance at 450 nm (OD450) after crystal violet staining (described below). According to Tavanti et al., biofilm-forming ability was categorized as follows: OD450 < 0.03, no biofilm; 0.03 ≤ OD450 < 0.08, weak biofilm; 0.08 ≤ OD450 < 0.16, moderate biofilm; OD450 ≥ 0.16, strong biofilm (Tavanti et al., 2010).

To assess biofilm biomass and metabolic activity at different temperatures, 100μL of the same cell suspension was added to each well of a 96-well plate (three replicates per strain) and incubated either at 37°C for 24 h or at 4°C for 14 days. Two different methods were then used to evaluate biofilm production (Pierce et al., 2008). For biomass quantification, 100 μL of 10% formaldehyde was added to each well to fix the biofilm at room temperature for 2 min. Then, 100μL of 20 mg/mL crystal violet solution was added to each well and incubated for 30 min. The wells were washed with 95% ethanol (100μL) to destain, and absorbance was measured at 620 nm using a spectrophotometer. For metabolic activity, an XTT reduction assay was performed: a solution of 0.5 mg/mL XTT and 1 mmol/L menadione was freshly prepared and 100μL of this mixture was added to each well, followed by incubation at 37°C for 2 h. The absorbance of the XTT formazan product was measured at 450 nm with a microplate reader.

### 2.3 Cell surface hydrophobicity test

The microbial adhesion to hydrocarbons (MATH) assay was used to evaluate cell surface hydrophobicity (CSH). *Candida* cells were cultured in Sabouraud dextrose broth at 37°C for 24 h and washed twice with PBS. The cells were resuspended in PBS and adjusted to an optical density (OD600) of 0.4–0.5 (initial OD, A0). Then 0.4 mL of n-hexadecane was gently layered over 3 mL of the cell suspension. The two-phase system was vortexed vigorously and allowed to separate, after which the OD600 of the aqueous phase was measured (A1). The percentage of cell surface hydrophobicity was calculated as: Hydrophobicity (%) = [1–(A1/A0)]×100% (Harjai et al., 2014). Each strain was tested in at least three independent experiments.

### 2.4 Adhesion assay

*Candida* isolates were grown on Sabouraud agar at 35°C for 48 h and washed twice with sterile PBS (10, 000 g, 5 min). The cells were resuspended in sterile PBS and adjusted to 2×10^6^ cells/mL. For the adhesion assay (Silva-Dias et al., 2015), 300μL of the yeast suspension was mixed with 300μL of fluorescent microspheres (2×10^8^ microspheres/mL) in a sterile tube and incubated on a shaker at 150 rpm at room temperature for 30 min. The mixture was then analyzed by flow cytometry, collecting 50, 000 yeast cell events per sample. According to Silva-Dias et al., the percentage of yeast cells with bound fluorescent microspheres (gated as population P2) was used as an indicator of adhesion ability. Adhesion was categorized as weak (P2 ≤ 20%), moderate (20% < P2 ≤ 30%), strong (30% < P2 ≤ 50%), or very strong (P2 > 50%). Additionally, the adhesion patterns were classified as homogenic or heterogenic based on the distribution of microspheres per cell: a homogenic distribution (single peak in flow cytometry) indicates each yeast cell is bound to only one microsphere, whereas a heterogenic distribution (multiple peaks) indicates individual yeast cells have more than one microsphere attached. All adhesion experiments were performed at least three times.

### 2.5 Colony and spore morphology observation

Each *Candida* isolate, after identification, was streaked for isolation on CHROMagar *Candida* medium and incubated at 35°C for 5 days to observe colony morphology. Smears of the colonies were then prepared and stained with Gram stain, lactophenol cotton blue, and a fungal fluorescence stain, to observe and record spore morphology.

### 2.6 Biofilm 1, 3-β-D-glucan release test

1, 3-β-D-glucan is a polysaccharide component widely present in fungal cell walls. The G-test (β-glucan test) uses a kinetic chromogenic assay to detect 1, 3-β-D-glucan. Given that serum 1, 3-β-D-glucan is an early indicator of invasive fungal infection, we explored the relationship between biofilm formation and 1, 3-β-D-glucan release by Candida isolates (Giacobbe et al., 2017). Each *Candida* strain was inoculated into five replicate wells of a 96-well plate (100μL of suspension per well at 2×10^6^ cells/mL) and incubated at 37°C for 24 h. The culture supernatants from the five wells were pooled and centrifuged (3000 rpm, 10 min). The samples were processed using a Fungitell 1, 3-β-D-glucan detection kit, and 1, 3-β-D-glucan concentrations were measured with a kinetic microplate reader at 75 minutes into the assay.

### 2.7 Neutrophil migration assay (transwell assay)

A Transwell chamber system was used to evaluate neutrophil chemotaxis. Neutrophils were isolated from fresh human whole blood using a neutrophil isolation kit and diluted in RPMI 1640 medium. Based on preliminary tests, we found that a neutrophil concentration of 5×10^5^/mL in the upper chamber and a *Candida* suspension of 5×10^4^ cells/mL in the lower chamber allowed clear observation of neutrophil migration through the membrane. Therefore, 200μL of the neutrophil suspension (5×10^5^/mL) was added to the upper chamber, and 500μL of a *Candida* culture (5×10^4^ cells/mL, grown for 24 h in RPMI 1640) was added to the lower chamber. After incubating at 37°C with 5% CO_2_ for 5 h, the Transwell insert was removed, and neutrophils that had migrated into the lower chamber were counted (Justus et al., 2023).

### 2.8 Antifungal susceptibility testing

Susceptibility to nine antifungal agents commonly used in ophthalmology was tested for each *Candida* isolate in both planktonic (free-living) and biofilm-forming states. The antifungal agents and concentration ranges were: amphotericin B (AMB, 0.125–16μg/mL), chlorhexidine (CDI, 0.03–16μg/mL), natamycin (NAT, 0.12–128μg/mL), terbinafine (TDN, 0.015–16μg/mL), voriconazole (VOR, 0.015–16μg/mL), posaconazole (POS, 0.015–16 μg/mL), itraconazole (ITR, 0.03–16μg/mL), and caspofungin (CAS, 0.016–16μg/mL). The MIC values of planktonic Candida were determined according to the CLSI standard protocols M27-S4 and M27-A3 (Clinical and Laboratory Standards Institute. (CLSI), 2008; Clinical and Laboratory Standards Institute. (CLSI), 2012). According to the experiment by Fernanda M. Bezerra et al (Bezerra et al., 2023), the *Candida* suspension concentration was adjusted to 0.5 McFarland and inoculated into 96-well cell culture plates (100μL/well), followed by incubation at 37°C for 24 h. A negative control group without antifungal drugs and a blank control group without *Candida* were established. The wells were rinsed three times with 200 μL PBS to remove non-adherent fungal cells. Antifungal drugs with serial two-fold dilutions were added and incubated at 35°C for 24 h, followed by three additional PBS washes (200 μL each) to eliminate non-adherent cells. Biofilm MICs (BMIC) were defined as the drug concentrations that reduced metabolic activity by 50% compared to drug-free controls. The metabolic activity of biofilms was assessed using the XTT reduction assay as previously described. Each drug-isolate combination was tested in triplicate, and mean values were calculated.

### 2.9 Microsatellite typing

Genomic DNA of *C. parapsilosis* isolates was extracted using a bacterial DNA extraction kit (Vazyme, Nanjing). Four polymorphic microsatellite loci (CP1, CP4, CP6, and B5) with high discriminatory power were selected based on the method of Sabino et al (Sabino et al., 2010). The primer sequences and fluorescent labels for these loci are shown in **Table 1** (Sabino et al., 2010). PCR amplification was performed in a 25 μL reaction containing 0.5 μL forward primer (10 μmol/L), 0.5 μL reverse primer (10 μmol/L), 1 μL DNA template, 0.5 μL dNTP mix (each 5 μmol/L), 2.5 μL 10× Taq buffer (with MgCl_2_), 0.2 μL Taq DNA polymerase (5 U/μL), and sterile water to 25 μL. The PCR cycling program was: 95°C for 5 min; 30 cycles of 94°C for 30 s, 55°C for 30 s, 72°C for 30 s; and a final extension at 72°C for 10 min. PCR products were analyzed by high-resolution capillary electrophoresis (Sangon Biotech, Shanghai). Allele sizes were determined for each locus, and multilocus genotypes were assigned based on the combination of alleles at the four loci. Allele and genotype frequencies were calculated using GeneMapper software.

**Table 1:** Microsatellite DNA sequences selected and primers used for PCR amplification.

Microsatellite designation	Primer sequence*^a^*	Size range size (bp)	Dye label
CP1	FWD:5’-AAAGTGCTACACACGCATCG-3’ REV: 5’-GGCTTGCAATTTCATTTCCT-3’	107-145	FAM
CP4	FWD:5’-CAAATCATCCAGCTTCAAACC-3’ REV:5’-CATCAAACAAGAATTCGATATCAC-3’	225-249	FAM
CP6	FWD: 5’-CAGGAACAGGACAATGGTGA-3’ REV: 5’-TCTGGAGCCTCTAGGACGTTT-3’	304-449	HEX
B5	FWD:5’-AGGTTTGTAGTAGTGTCCCTATGG-3’ REV: 5’-TATCTCTCTCGCCATTTGAACG-3’	228-303	HEX

^a^ FWD, forward primer; REV, reverse primer.

### 2.10 Data analysis

GraphPad Prism 10 (GraphPad Software, San Diego, CA) was used for statistical analyses and graphing. Quantitative data are expressed as mean ± standard error of the mean (Mean ± SEM). For non-normally distributed data, the Kruskal-Wallis test was used to compare groups, and *p < 0.05* was considered statistically significant. Correlations were analyzed using Pearson’s correlation coefficient or Spearman’s rank correlation coefficient, as appropriate. For microsatellite data, cluster analysis of *C. parapsilosis* genotypes was performed using the UPGMA method in MVSP (v3.13n) software, and genetic distances between isolates were calculated.

## 3 Result

### 3.1 Strain distribution

A total of 45 *Candida* isolates from postoperative ocular infections were identified by MALDI-TOF MS. These included 22 C*. parapsilosis* (48.9%), 16 C*. albicans* (35.6%), 5 C*. tropicalis* (11.1%), and 2 C*. glabrata* (4.4%). All identification scores were ≥ 2.000, indicating reliable species-level identification.

### 3.2 Epidemiological characteristics of infected patients

Among the 45 patients with postoperative ocular *Candida* infection, 26 (57.8%) were male and 19 (42.2%) were female. The mean age was 51.1 ± 14.8 years. Fourteen patients (31.1%) were from Northeast China, 27 (60.0%) were from North China, and 4 (8.9%) were from other regions. The most common underlying systemic disease was type 2 diabetes mellitus (22.2%, 10/45), followed by hypertension (13.3%, 6/45). The most frequent ophthalmic surgery associated with *Candida* infection was corneal transplantation (33.3%, 15/45), followed by cataract surgery (24.4%, 11/45) and vitrectomy (15.6%, 7/45). We grouped the patients by the infecting *Candida* species (*C. parapsilosis* group, *C. albicans* group, *C.* tropicalis group, *C. glabrata* group) and analyzed the types of prior ocular surgery in each group. Corneal transplantation was the most common surgery in the *C. parapsilosis* group (54.5%, 12/22), while cataract surgery was most common in the *C. albicans* group (31.3%, 5/16). Due to the small case numbers, the predominant surgery type in the *C. tropicalis* and *C. glabrata* groups was not clearly determined. In terms of clinical outcomes, 25 patients (55.6%) had their infections controlled with medication alone, whereas 20 patients (44.4%) required therapeutic corneal transplantation. Notably, in the *C. parapsilosis* group, 72.7% (16/22) of patients ultimately required surgical intervention, and 3 patients (13.6%) underwent a repeat corneal transplant (**Table 2**).

**Table 2:** Population characteristics of *Candida* keratitis cases.

Variate	*Candida parapsilosis* (n=22)	*Candida albicans* (n=16)	*Candida tropicalis* (n=5)	*Candida glabrata* (n=2)
Age, years	53.2 ± 15.2	57.4 ± 15.7	56.8 ± 10.6	57 ± 12.7
Male	13 (59.1)	10 (62.5)	3 (60)	-
Systemic disease				
Diabetes	6 (27.3)	3 (18.8)	1 (20)	-
High blood pressur	2 (9.1)	4 (25)	-	-
Renal insufficiency	-	-	1 (20)	
Autoimmune diseases	1 (4.5)	-	-	1 (50)
Neoplastic diseases	1 (4.5)	-	-	-
Types of Ocular surgery				
Keratoplasty	12 (54.5)	2 (12.5)	1 (20)	-
Cataract surgery	5 (22.7)	5 (31.3)	1 (20)	-
Vitrectomy	2 (9.1)	4 (25)	1 (20)	-
Glaucoma surgery	1 (4.5)	2 (12.5)	-	-
Amnioplasty	1 (4.5)	-	-	-
Other ocular surgery	2 (9.1)	4 (25)	2 (40)	2 (100)
Management				
Medicine	6 (27.3)	13 (81.3)	4 (80)	2 (100)
Surgery	16 (72.7)	3 (18.8)	1 (20)	-

### 3.3 Biofilm formation ability, biomass, and metabolic activity at different temperatures

**Figure 1:**
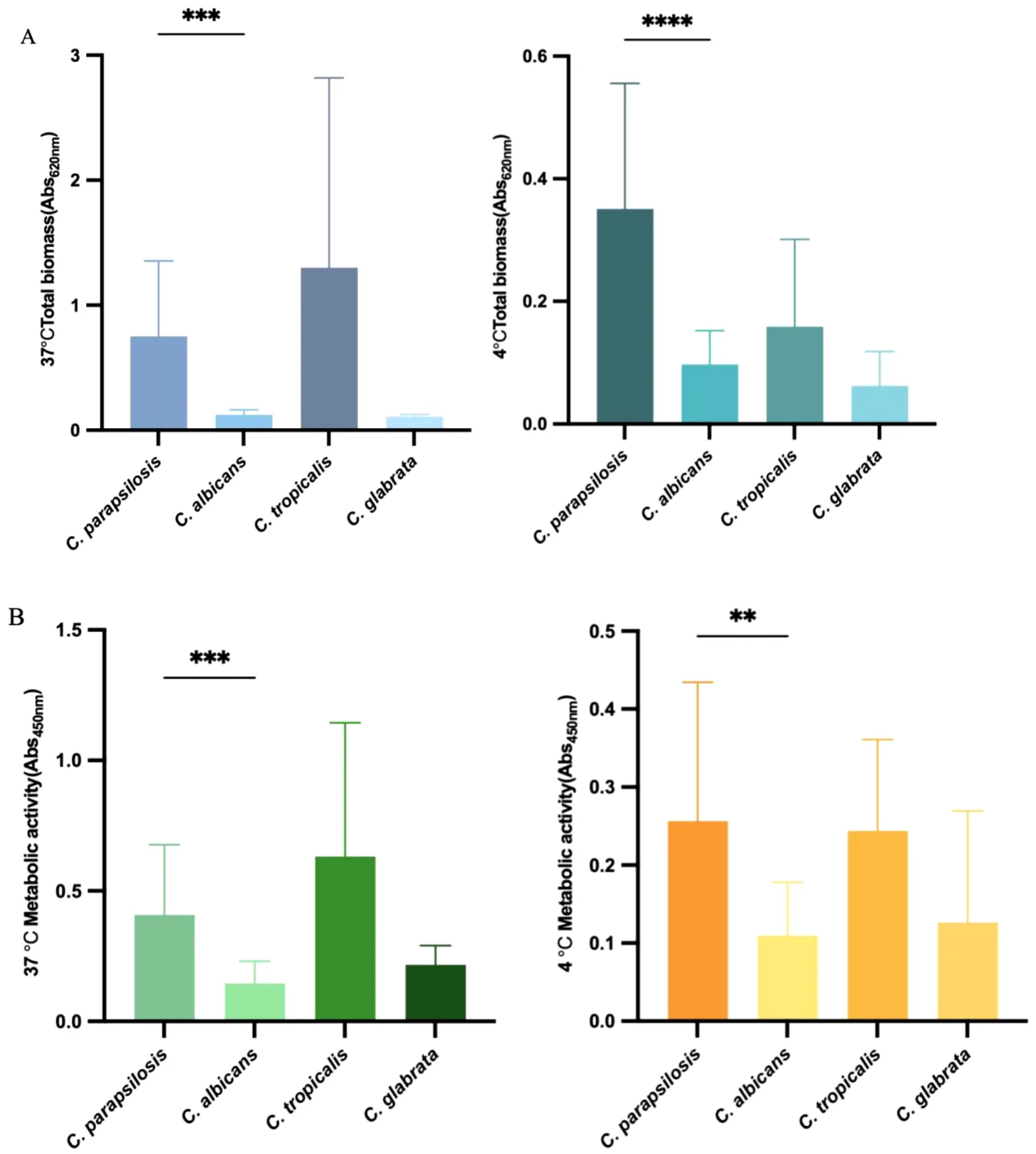
Biofilm Formation Ability, Biomass, and Metabolic Activity at Different Temperatures. **(A)** Crystal violet staining assay measures different *Candida* species’ biomass at 37°Cand 4°C. **(B)** XTT assay measures different *Candida* species’ biofilm metabolic activity at 37°Cand 4°C. Error bars represent the standard deviation among results for different isolates.Each isolate was tested for its ability to form biofilm at least 3 times. ****p < 0.0001; ***p < 0.001; **p < 0.01.

*C. parapsilosis* and *C. tropicalis* exhibited significantly higher biofilm-forming ability than *C. albicans* and *C. glabrata* (*p < 0.001*). Among all 45 isolates, 18 (40.0%) were classified as strong biofilm formers (OD450 ≥ 0.16), 6 (13.3%) as weak biofilm formers (OD450 < 0.08), and 21 (46.7%) as non-biofilm-forming. As shown in **Figure 1**, at both 37°C and 4°C, the total biofilm biomass and metabolic activity of *C. parapsilosis* biofilms were significantly higher than those of *C. albicans*(*p<0.001, p<0.0001*)(*p<0.001, p<0.01*). At 37°C, the biofilm biomass of *C. parapsilosis* was positively correlated with its biofilm metabolic activity (r=0.807**), whereas no such correlation was observed for the other species. At 4°C, there was no correlation between biofilm biomass and metabolic activity for any of the *Candida* species.

### 3.4 Cell surface hydrophobicity

**Figure 2:**
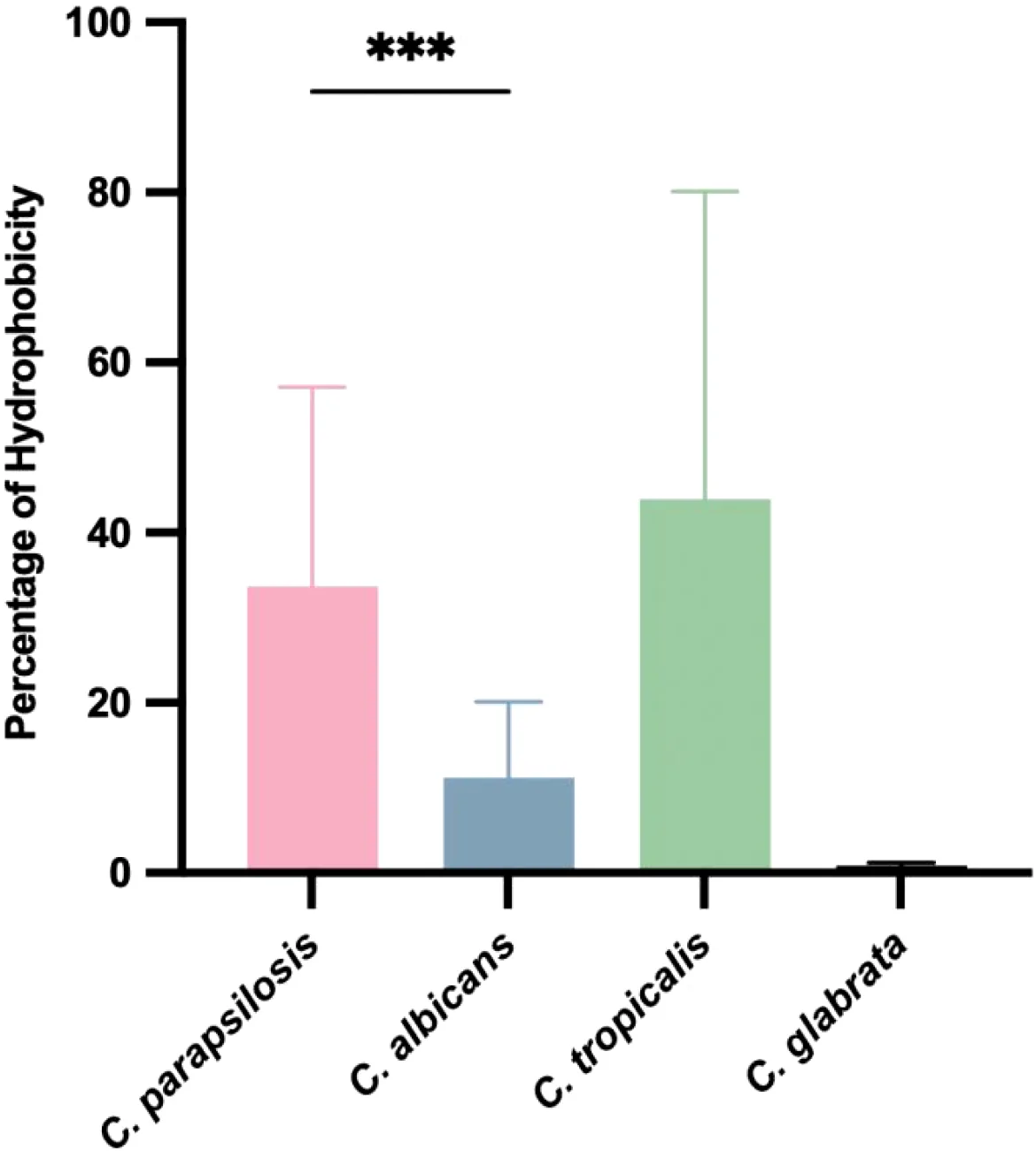
*Candida* hydrophobicity was measured according MATH test. Results are representative of the mean results of each species. Each strain was tested three times.There was no significant comparison among other strains. ***p < 0.001.

*Candida parapsilosis* and *C. tropicalis* showed significantly higher cell surface hydrophobicity percentages than *C. albicans* and *C. glabrata* (*p < 0.001*) (**Figure 2**). In *C. parapsilosis* and *C. tropicalis*, the cell surface hydrophobicity was positively correlated with biofilm biomass (r=0.48* and r=0.90*), whereas no correlation was observed for *C. albicans* or *C. glabrata*.

### 3.5 Adhesion assay

**Figure 3:**
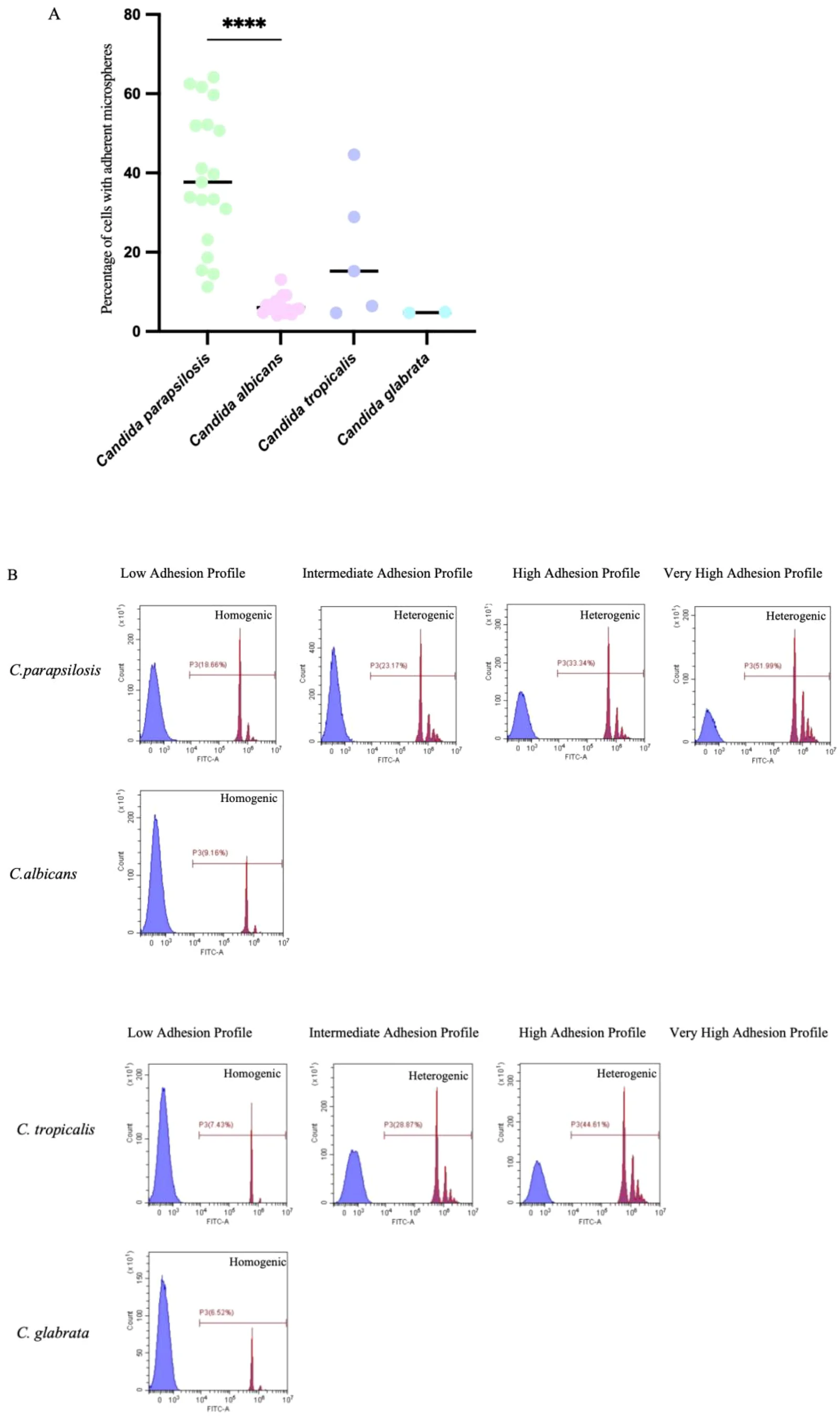
Representation of *Candida* adhesion profiles. **(A)** The species with higher percentage of cells with adherent microspheres are *C. parapsilosis*. Results represent the mean of at least 3 independent experiments, performed in triplicate. ****p < 0.0001. **(B)** Homogenic (a homogenous distribution pattern characterizes a population of yeast cells bound to the same number of microspheres, frequently binding to a single microsphere) and Heterogenic (a heterogeneous pattern displays the presence of different peaks beyond the third logarithmic decade and indicates that more than a single microsphere is attached to each yeast cell) distribution patterns.P3:Percentage of cell with adherent microspheres.

*C. parapsilosis* demonstrated the strongest adhesion ability among the species, with an adhesion percentage of (38.54 ± 3.97)%, and 73.7% of *C. parapsilosis* isolates were classified as having strong or very strong adhesion. *C. tropicalis* had the second highest adhesion (19.98 ± 7.50%), with 20% of isolates classified as strong/very strong. *C. albicans* and *C. glabrata* exhibited weak adhesion, with adhesion percentages below 10%. The adhesion of *C. parapsilosis* was significantly higher than that of *C. albicans* (*p < 0.0001*). A heterogeneous adhesion distribution pattern (multiple peaks in flow cytometry) was observed in 63.2% of *C. parapsilosis* isolates, which was significantly higher than in the other species (**Figure 3**). Additionally, *C. parapsilosis* adhesion was positively correlated with biofilm biomass (r=0.60**), a correlation significantly stronger than that of the other species. *C. albicans* also showed a positive correlation between adhesion and biofilm biomass (r=0.56*), as did *C. tropicalis* (r=0.90*).

### 3.6 Colony and spore morphology records

**Figure 4:**
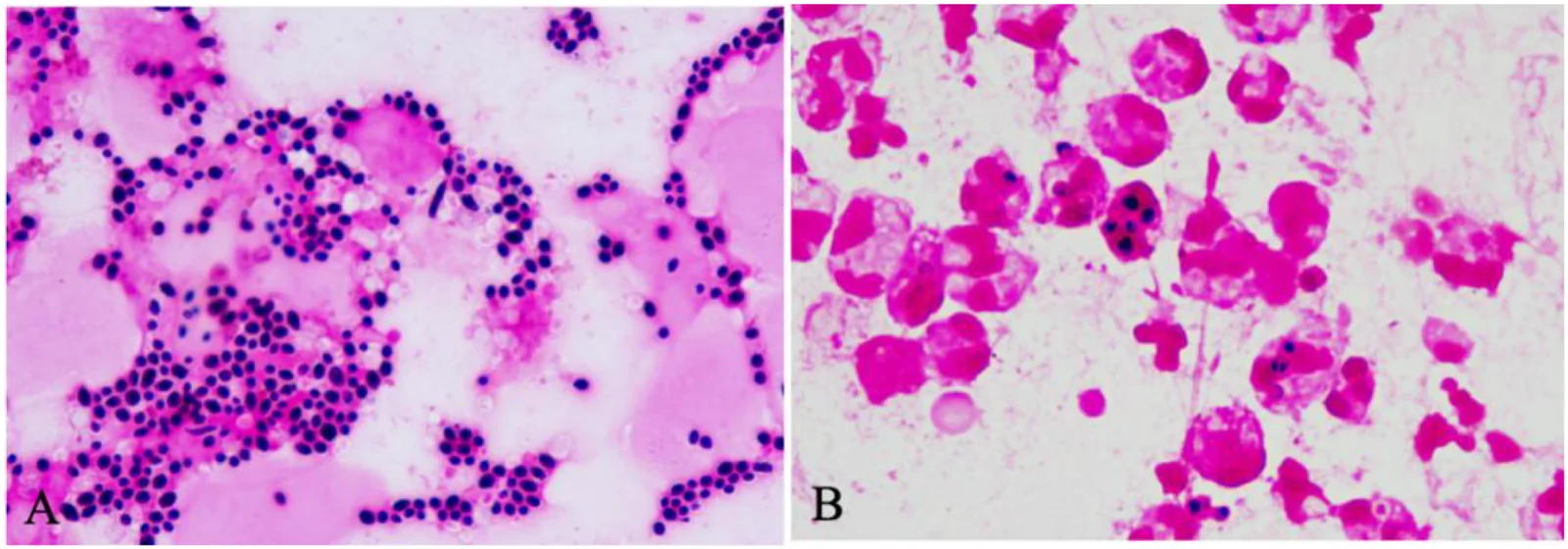
Differences under the microscope (Gram stain, 1000×) between corneal exudates infected with biofilm-producing vs. non-biofilm-producing *C. parapsilosis*. **(A)** Corneal surface exudate with a biofilm-producing strain: numerous fungal spores (purple) attached to the surfaces of apoptotic cells and in the intercellular spaces. **(B)** Corneal surface exudate with a non-biofilm-producing strain: few extracellular fungal spores are observed; most spores have been phagocytosed by neutrophils and mononuclear phagocytes.

In preliminary microscopic examinations of clinical corneal exudate smears, we observed that *Candida* could appear in two forms. In one form (non-biofilm-forming *Candida*), fungal spores were distributed sparsely and could be phagocytosed by neutrophils; in the other form (biofilm-producing *Candida*), fungal spores appeared in sheets that neutrophils could not phagocytose (**Figure 4**). This finding corroborated that some *Candida* can form biofilms upon infecting donor corneas.

**Figure 5:**
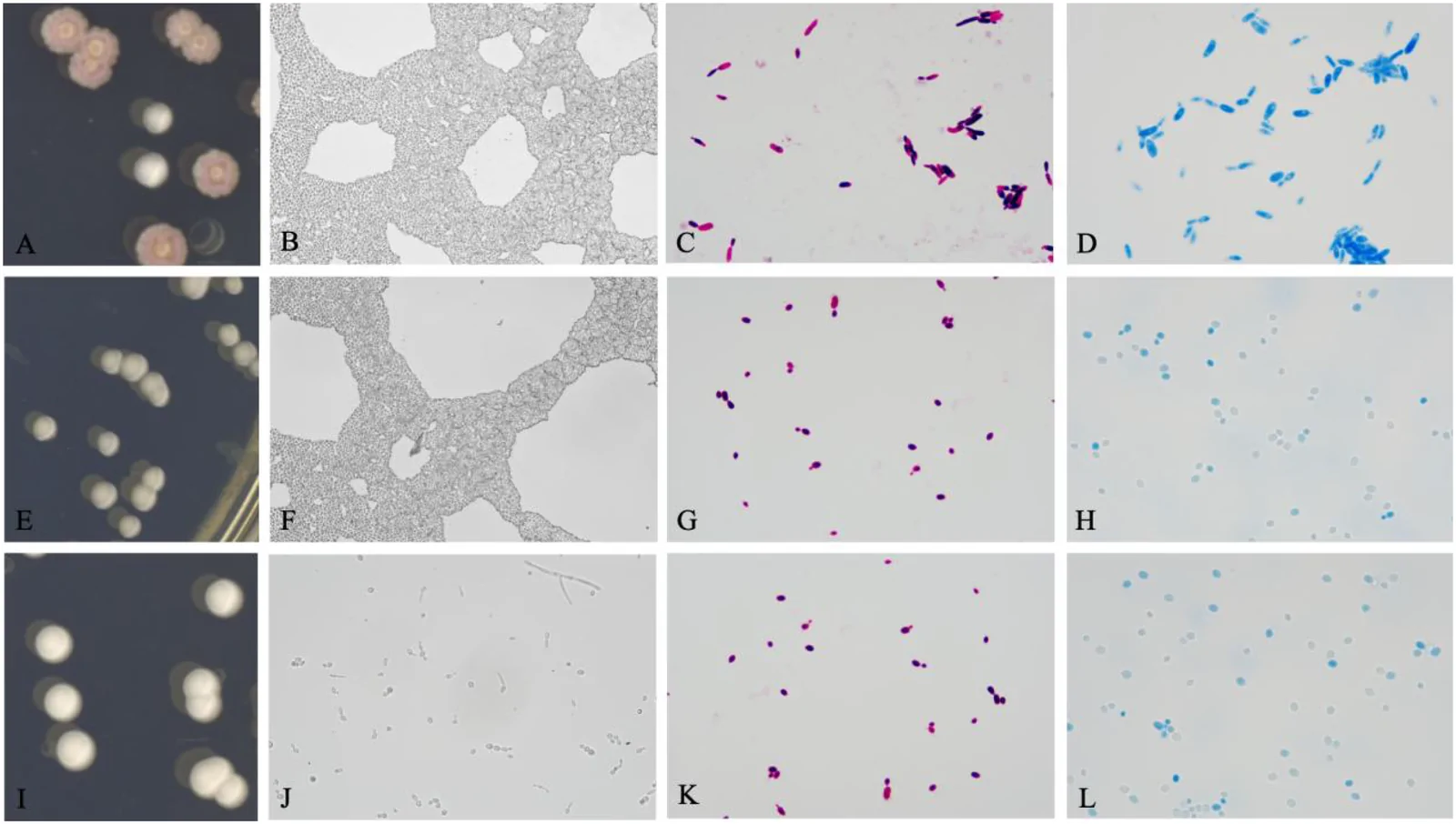
Relationship of biofilm-forming ability with colony morphology, spore morphology, and staining characteristics. **(A)** Colony of a strong biofilm-forming strain (*C. parapsilosis*, rough morphotype). **(B)** Robust biofilm formed by a strong biofilm-forming strain. **(C)** Gram-stained spores of a strong biofilm-forming *Candida* showing pseudohyphal morphology. **(D)** Lactophenol cotton blue-stained spores of a strong biofilm-forming *Candida* showing uniform, deep staining. **(E)** Colony of a weak biofilm-forming strain (*C. albicans*, smooth morphotype). **(F)** Weak biofilm formed by a weak biofilm-forming strain. **(G)** Gram-stained spores of a weak biofilm-forming *Candida* showing ovoid yeast morphology. **(H)** Lactophenol cotton blue-stained spores of a weak biofilm-forming *Candida* showing variable staining intensity. **(I)** Colony of a non-biofilm-forming strain (*C. glabrata*, smooth morphotype). **(J)** Microscopic appearance of a non-biofilm-forming strain (no biofilm observed). **(K)** Gram-stained spores of a non-biofilm-forming *Candida*. **(L)** Lactophenol cotton blue-stained spores of a non-biofilm-forming *Candida*. Biofilm images 400×; Gram stain 1000×; lactophenol cotton blue 1000×.

After 5 days of culture on CHROMagar, the colony and spore morphologies of the *Candida* isolates were recorded and compared based on their biofilm-forming abilities (**Figure 5**). We found that isolates with strong biofilm-forming ability had rough, wrinkled and dry colonies. On Gram staining, these isolates showed spores in pseudohyphal form, and on lactophenol cotton blue staining their spores were uniformly and deeply stained. In contrast, isolates with weak biofilm-forming ability had smooth, moist colonies; on Gram stain their spores appeared as oval yeasts, and on lactophenol cotton blue their spores showed uneven staining intensity.

### 3.7 Relationship between biofilm formation and 1, 3-β-D-glucan release

**Figure 6:**
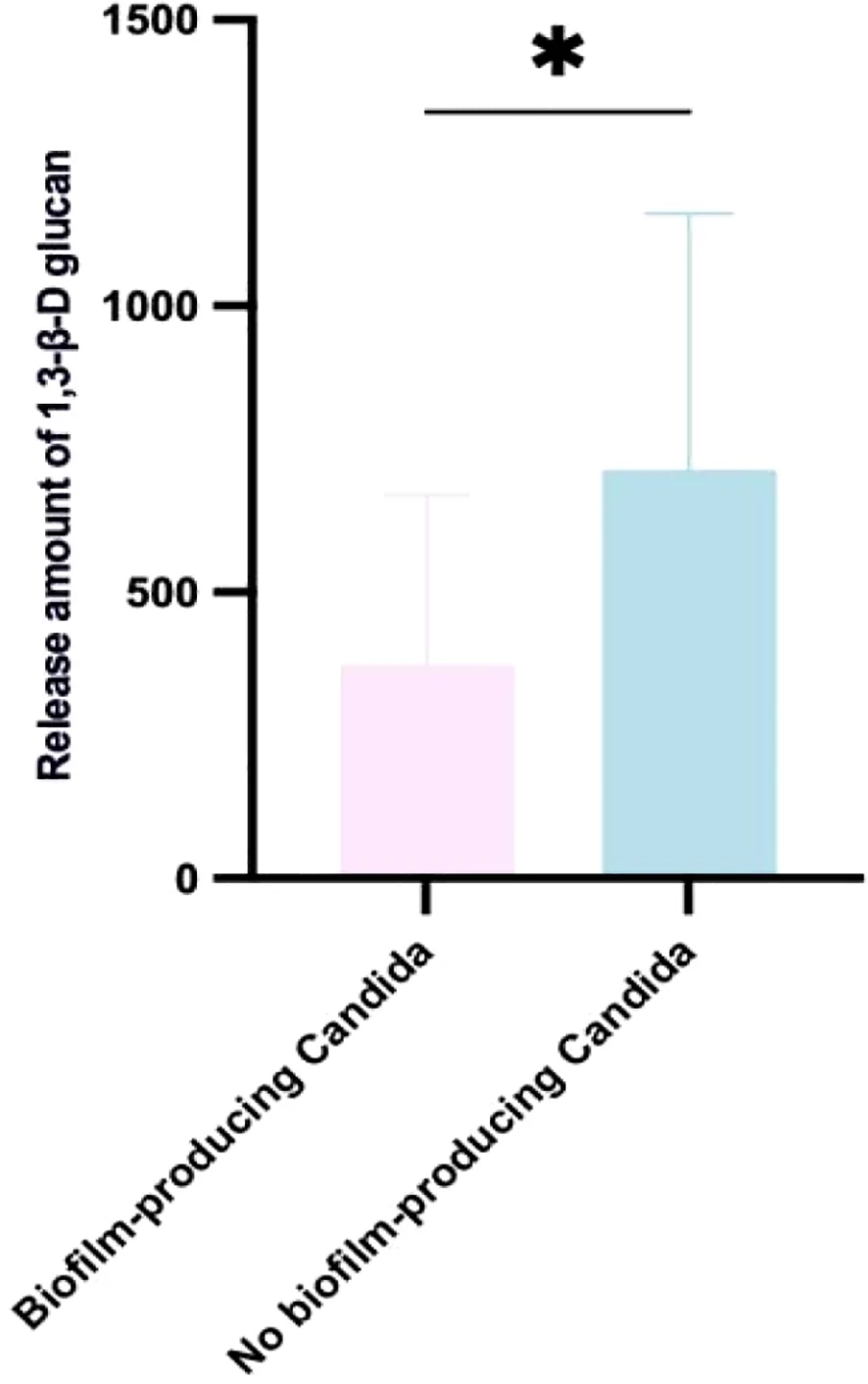
Relationship between biofilm formation and 1, 3-β-D-glucan release. Non-biofilm-forming *Candida* strains released significantly more 1, 3-β-D-glucan than strong biofilm-forming strains. Each experiment was performed in triplicate. *p < 0.05.

Our results showed that *Candida* strains with strong biofilm formation released 1, 3-β-D-glucan at 374.2 ± 295.8 pg/mL, whereas non-biofilm-forming strains released 714.3 ± 447.0 pg/mL (**Figure 6**). The non-biofilm group released significantly more 1, 3-β-D-glucan than the strong biofilm group (*p < 0.05*), indicating that non-biofilm-forming *Candida* strains exhibit higher antigenicity. Moreover, the amount of 1, 3-β-D-glucan released was negatively correlated with the biofilm’s metabolic activity (r = –0.64*).

### 3.8 Neutrophil migration experiment

**Figure 7:**
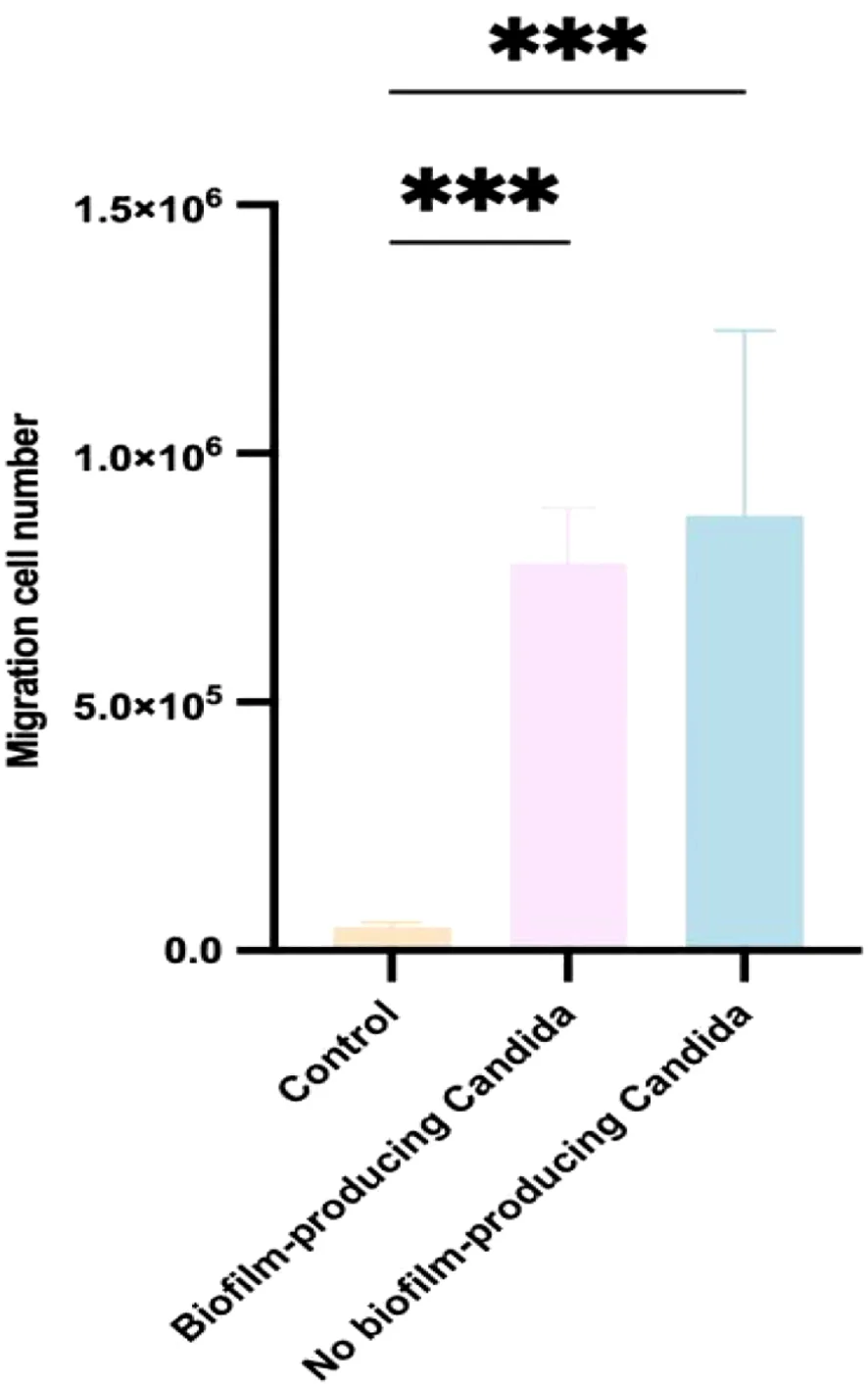
Neutrophil migration (Transwell) assay for *Candida* strains with different biofilm-forming abilities. Non-biofilm-forming strains (which release higher levels of 1, 3-β-D-glucan) attracted more neutrophils than biofilm-forming strains. Each experiment was performed in triplicate. ***p < 0.001.

In the Transwell chemotaxis assay, the control group (no *Candida*) induced the migration of 4.8×10^4^ ± 8.4×10^3^ neutrophils. The high biofilm-producing group induced 7.8×10^5^ ± 1.1×10^5^ neutrophils to migrate, while the non-biofilm-producing group induced 8.76×10^5^ ± 3.7×10^5^ neutrophils (**Figure 7**). In the non-biofilm group, one *C. parapsilosis* isolate attracted as many as 1.49×10^6^ neutrophils; this strain’s 1, 3-β-D-glucan level was 410.2 pg/mL, without a notably elevated antigenic response.

### 3.9 Antifungal susceptibility of biofilm and planktonic *Candida*

Planktonic antifungal susceptibility tests were successfully performed on all 45 *Candida* isolates. However, due to disruption of biofilms during the washing steps for weak biofilm producers, only 18 strong biofilm-forming isolates (15 C*. parapsilosis* and 3 C*. tropicalis*) were evaluated for antifungal susceptibility in the biofilm state. Except for *C. tropicalis* (which had terbinafine, voriconazole, posaconazole, and itraconazole MICs > 16 μg/mL in both planktonic and biofilm states), all *Candida* species showed markedly higher MICs in the biofilm state than in the planktonic state. Across all isolates, caspofungin maintained low MIC values in both states with no significant differences. The planktonic MIC results for all isolates are presented in **Table 3**, and the biofilm MIC (BMIC) results for the 18 strong biofilm-forming isolates are presented in **Table 4**.

**Table 3:** Antifungal susceptibilities of different *Candida* strains under planktonic growth conditions.

*Candida* species	Planktonic MIC (μg/mL) of:								CAS^i^
	AMB^a^	CDI^b^	NAT^c^	TDN^d^	VOR^e^	POS^f^	ITR^g^	FLU^h^	
*C. parapsilosis* ATCC 22019	2	1	8	4	0.015, S	0.25	0.25	0.5	0.5, S
*C. parapsilosis* 2407	1	1	8	4	0.015, S	0.03, S	0.06	0.25	0.5, S
*C. parapsilosis* 1951	0.25	2	8	4	0.03, S	0.03, S	0.06	0.5	0.25, S
*C. parapsilosis* 2699	0.25	2	8	2	0.03, S	0.03, S	0.03	0.5	0.25, S
*C. parapsilosis* 2132	0.5	1	4	2	0.015, S	0.03, S	0.03	0.25	0.5, S
*C. parapsilosis* 1167	0.5	2	8	4	0.015, S	0.03, S	0.06	0.25	0.5, S
*C. parapsilosis* 1277	0.25	2	8	2	0.03, S	0.03, S	0.03	0.5	0.25, S
*C. parapsilosis* 1354	0.5	2	4	2	0.015, S	0.03, S	0.06	0.25	0.5, S
*C. parapsilosis* 1513	0.5	2	8	4	0.03, S	0.03, S	0.06	0.25	0.5, S
*C. parapsilosis* 1514	0.25	1	4	2	0.015, S	0.03, S	0.03	0.25	0.5, S
*C. parapsilosis* 389	0.25	1	8	4	0.06, S	0.06, S	0.125	0.5	0.25, S
*C. parapsilosis* 1037	0.5	1	8	2	0.03, S	0.06, S	0.125	0.25	0.5, S
*C. parapsilosis* 1348	0.5	2	8	4	0.015, S	0.03, S	0.06	0.25	0.5, S
*C. parapsilosis* 1350	0.5	2	8	4	0.03, S	0.03, S	0.06	0.25	0.5, S
*C. parapsilosis* 2478	0.5	2	8	4	0.015, S	0.03, S	0.06	0.25	0.5, S
*C. parapsilosis* 2581	0.5	2	4	2	0.015, S	0.03, S	0.06	0.25	0.5, S
*C. parapsilosis* 2591	0.5	2	8	4	0.03, S	0.03, S	0.06	0.25	0.5, S
*C. parapsilosis* 2229	0.5	2	8	4	0.03, S	0.03, S	0.06	0.25	0.5, S
*C. parapsilosis* 2766	0.25	1	4	2	0.015, S	0.03, S	0.03	0.25	0.5, S
*C. parapsilosis* 1194	0.5	2	8	4	0.015, S	0.03, S	0.06	0.25	0.5, S
*C. parapsilosis* 1192	0.25	2	8	2	0.03, S	0.03, S	0.03	0.5	0.25, S
*C. parapsilosis* 1207	0.5	2	8	4	0.015, S	0.03, S	0.06	0.25	0.5, S
*C. albicans* 1115	0.25	2	4	>16	0.125, S	0.06, S	0.125	4	0.064, S
*C. albicans* 1862	0.5	2	4	>16	0.015, S	0.03, S	0.03	0.5	0.25, S
*C. albicans* 1868	0.125	2	4	>16	0.015, S	0.03, S	0.03	0.5	0.064, S
*C. albicans* 1928	0.125	1	8	>16	0.015, S	0.015, S	0.03	0.05	0.125, S
*C. albicans* 2611	0.25	1	4	>16	0.03, S	0.06, S	0.06	0.05	0.125, S
*C. albicans* 1480	0.125	1	8	>16	0.015, S	0.015, S	0.03	0.25	0.064, S
*C. albicans* 2695	0.25	1	8	>16	0.015, S	0.015, S	0.03	0.25	0.125, S
*C. albicans* 1213	0.25	2	4	>16	0.015, S	0.015, S	0.03	0.25	0.064, S
*C. albicans* 1297	0.25	2	8	>16	0.015, S	0.015, S	0.06	0.25	0.125, S
*C. albicans* 1372	0.125	1	8	>16	0.03, S	0.06, S	0.06	1	0.064, S
*C. albicans* 1519	0.25	1	4	>16	0.015, S	0.06, S	0.06	1	0.125, S
*C. albicans* 1549	0.25	2	4	>16	0.03, S	0.015, S	0.06	0.25	0.064, S
*C. albicans* 1770	0.125	1	8	>16	0.03, S	0.06, S	0.03	0.25	0.064, S
*C. albicans* 3137	0.25	1	8	>16	0.015, S	0.015, S	0.03	1	0.064, S
*C. albicans* 1966	0.25	1	8	>16	0.03, S	0.06, S	0.03	0.25	0.064, S
*C. albicans* 1503	0.125	2	4	>16	0.015, S	0.06, S	0.06	0.25	0.064, S
*C. tropicalis* 2099	0.25	1	4	>16	>16, R	>16, R	>16	4	0.25, S
*C. tropicalis* 2098	0.25	1	4	>16	>16, R	>16, R	>16	4	0.25, S
*C. tropicalis* 2491	0.25	1	8	>16	>16, R	>16, R	>16	4	0.25, S
*C. tropicalis* 2379	0.25	1	4	>16	>16, R	>16, R	>16	4	0.25, S
*C. tropicalis* 2800	0.25	1	4	>16	>16, R	>16, R	>16	4	0.25, S
*C. glabrata* 2555	0.25	1	4	2	0.015, S	0.015, S	0.03	0.25	0.064, S
*C. glabrata* 2105	0.25	1	4	2	0.015, S	0.015, S	0.03	0.25	0.064, S

^a^ AMB, amphotericinB;

^b^ CDI, Chlorhexidine;

^c^ NAT, natamycin;

^d^ TDN, terbinafine;

^e^ VOR, Voriconazole;

^f^ POS, Posaconazole;

^g^ ITR, Itraconazole;

^h^ FLU, Fluconazole;

^i^ CAS, Caspofungin

**Table 4:** Antifungal susceptibilities of different *Candida* strains under biofilm(BMIC) growth conditions.

*Candida* species	BMIC50 MIC (μg/mL) of:								CAS
	AMB	CDI	NAT	TDN	VOR	POS	ITR	FLU	
*C. parapsilosis* 2407	>16	>16	>128	>16	>16	>16	>16	>128	2
*C. parapsilosis* 1951	>16	>16	>128	>16	>16	>16	>16	>128	0.125
*C. parapsilosis* 2699	>16	>16	>128	>16	>16	>16	>16	>128	0.25
*C. parapsilosis* 2132	>16	>16	>128	>16	>16	>16	>16	>128	0.125
*C. parapsilosis* 389	8	>16	64	8	8	>16	>16	>128	0.016
*C. parapsilosis* 1037	>16	>16	>128	>16	>16	>16	>16	>128	0.125
*C. parapsilosis* 1348	16	>16	>128	>16	>16	>16	>16	>128	0.016
*C. parapsilosis* 1350	8	16	32	>16	>16	>16	>16	>128	0.5
*C. parapsilosis* 2478	>16	>16	128	>16	>16	>16	>16	>128	0.016
*C. parapsilosis* 2581	>16	>16	>128	>16	>16	>16	>16	>128	0.5
*C. parapsilosis* 2591	8	4	32	>16	>16	>16	8	>128	0.032
*C. parapsilosis* 2766	>16	>16	>128	>16	>16	>16	>16	>128	0.125
*C. parapsilosis* 1194	>16	>16	>128	>16	>16	>16	>16	>128	0.125
*C. parapsilosis* 1192	>16	>16	128	>16	>16	>16	>16	>128	0.016
*C. parapsilosis* 1207	>16	>16	>128	>16	>16	>16	>16	>128	0.5
*C. tropicalis* 2099	>16	>16	>128	>16	>16	>16	>16	>128	0.064
*C. tropicalis* 2098	>16	>16	>128	>16	>16	>16	>16	>128	0.5
*C. tropicalis* 2491	>16	>16	>128	>16	>16	>16	>16	>128	0.125

### 3.10 Microsatellite genotyping

**Figure 8:**
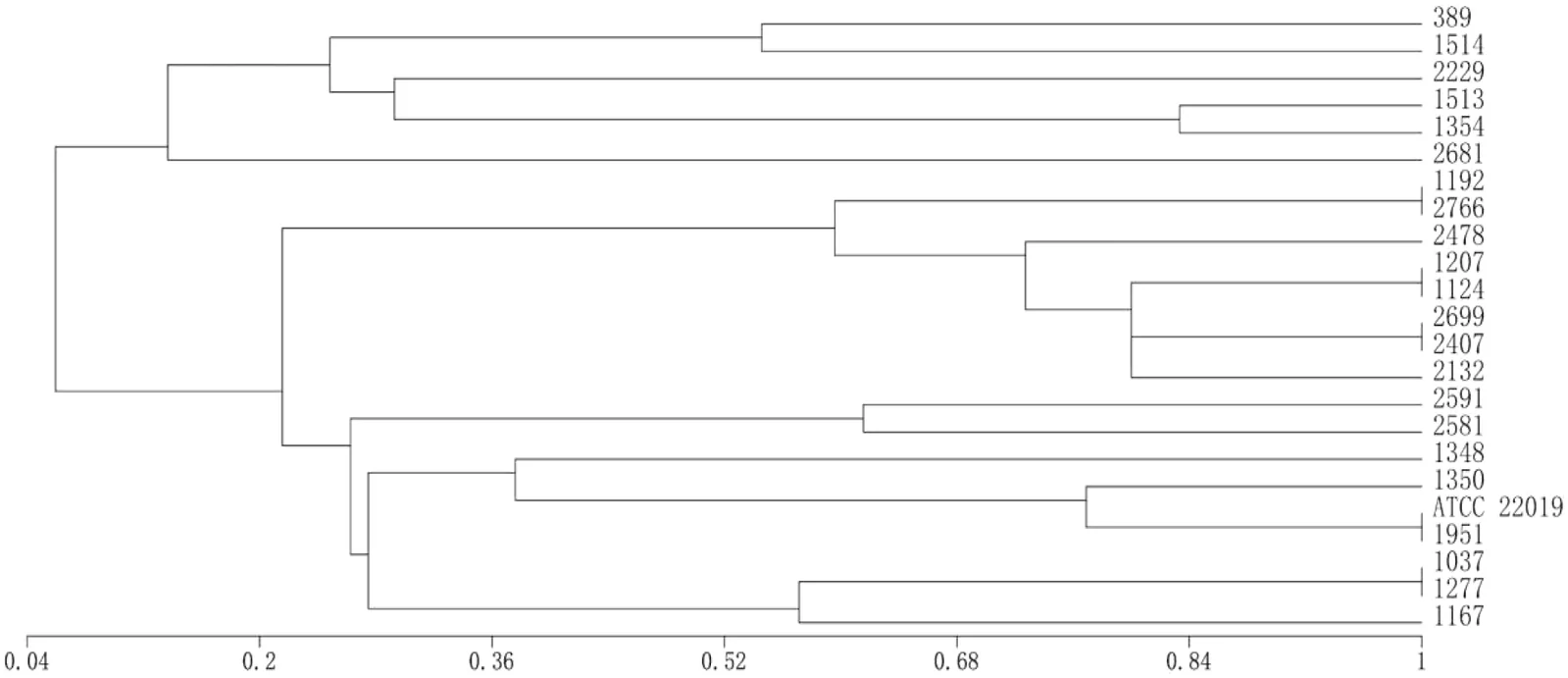
Dendrogram showing clustering of *Candida parapsilosis* isolates obtained from corneal transplant status, based on microsatellite multilocus genotyping. Genetic distances were calculated by using MVSP (v3.13n) software program and clustering performed by using UPGMA method. *Candida parapsilosis* ATCC 22019 belongs to type II.

Microsatellite multilocus typing results for the 22 C*. parapsilosis* isolates are shown in **Table 5**. The 22 isolates were resolved into 18 distinct genotypes. Types I, II, III, and IV each contained two isolates with identical allele profiles, while each of the remaining genotypes was represented by a single isolate, indicating a high genetic diversity among the isolates(**Figure 8**). Notably, all isolates from the year 2024 were grouped into genotype III or IV, and all of these were high biofilm-producing strains. This suggests a potential clustering trend for *C. parapsilosis* genotypes in 2024.

**Table 5:** Microsatellite multilocus analysis of *Candida parapsilosis* isolates obtained from eye surgery.

*Candida* species	Typing	Date	Multilocus genotype							
			CP1		CP4		CP6		B5	
2699	I	2014	129	129	243	243	448	448	231	270
2407		2010	129	129	243	243	448	448	231	270
1037	II	2018	113	129	240	249	406	406	264	288
1277		2016	113	129	243	243	358	406	264	288
1194	III	2024	129	129	242	242	473	473	230	271
1207		2024	129	129	242	242	473	473	230	271
2766	IV	2024	129	129	243	243	358	445	246	270
1129		2024	129	129	243	243	358	445	246	270
1951	V	2012	113	113	225	240	364	364	267	270
2681	VI	2012	143	145	240	240	304	304	276	279
2132	VII	2014	129	129	243	243	358	358	231	270
1167	VIII	2014	113	127	243	243	358	406	264	288
1354	IX	2016	107	119	237	237	304	319	285	288
1513	X	2016	119	119	237	237	304	304	285	288
1514	XI	2018	127	127	237	237	304	415	234	234
389	XII	2018	127	127	237	237	415	454	276	291
1348	XIII	2019	129	129	240	240	364	367	300	303
1350	XIV	2019	113	129	240	243	364	364	267	270
2478	XV	2022	129	129	243	243	364	364	268	270
2581	XVI	2023	129	129	243	243	373	445	231	270
2591	XVII	2023	127	127	240	240	322	406	228	270
2229	XVIII	2023	107	107	237	237	322	322	270	270

## 4 Discussion

1. Despite many reports on *C. parapsilosis* causing bloodstream and other infections, studies on its ocular pathogenic risk factors are relatively few. Our study showed that *C. parapsilosis* is the most prevalent *Candida* species in postoperative ocular infections, and the rate of infection after corneal transplantation is significantly higher for *C. parapsilosis* than for the other three *Candida* species, similar to the findings of Tanya Trinh et al (Thareja et al., 2020). Corneal transplantation provides *C. parapsilosis* a route to infect ocular tissue, making postoperative infection more likely. Donor corneas share many properties with indwelling medical devices – for example, a smooth surface, tensile strength, supportiveness, and good biocompatibility – which also make them conducive to *C. parapsilosis* colonization. Our study also found that a high proportion of patients with postoperative ocular *Candida* infection had type 2 diabetes, a condition that impairs immune function and creates a hyperglycemic environment in which *C. parapsilosis* can more readily form biofilms, leading to pan-corneal infection and dissemination (Dorko et al., 2005; Hernández-Pabón et al., 2024). In our series, 72.7% of patients with *C. parapsilosis* infections required surgical intervention (e.g., therapeutic keratoplasty), a rate markedly higher than for the other *Candida* species. This may be because biofilm formation by *C. parapsilosis* renders medical (drug) therapy less effective, necessitating surgical management.

2. A greater proportion of *C. parapsilosis* isolates produced biofilm compared to *C. tropicalis*, *C. albicans*, and *C. glabrata*. Nearly half of the high biofilm-producing isolates in post-corneal transplant infections were *C. parapsilosis*. Although at 37°C *C. parapsilosis* biofilms did not have as high a biomass or metabolic activity as those of *C. tropicalis*, the biofilm production among *C. tropicalis* isolates varied widely. At 4°C, *C. parapsilosis* had a significantly greater ability to form biofilm than the other species. These characteristics indicate that *C. parapsilosis* not only generally has a stronger capacity to form biofilms, but is also better adapted to low-temperature environments. Donor corneas are stored at 4°C in Optisol-GS for 10–14 days; low temperature favors *C. parapsilosis* biofilm formation, which may be one reason *C. parapsilosis* infection rates after corneal transplantation are higher than with other species. *C. tropicalis* also showed a high biofilm-forming ability at 37°C, but did not have an advantage at 4°C.

3. Adhesion is a crucial initial step in *Candida* pathogenesis and is considered an important virulence factor (de Souza et al., 2023). Studies have shown that *C. parapsilosis* has a higher adhesion to biomaterials than other *Candida* species (Silva et al., 2011; Borges et al., 2018). This is closely related to the cell wall composition; in the yeast form, *C. parapsilosis* can express more adhesin proteins such as Als1, Als3, Als6, Als7, and Hwp1 (Cuéllar-Cruz et al., 2012). Although some research suggests that adhesion and biofilm production are not directly correlated (Silva et al., 2010), our findings indicate that in postoperative corneal *Candida* infections, biofilm biomass is positively correlated with adhesion strength for most species (except *C. glabrata*). Specifically, only *C. parapsilosis* showed a strong correlation between biofilm biomass and metabolic activity at 37°C, perhaps because each *Candida* species has its own metabolic rate that may not directly reflect biofilm mass. We also found that *C. parapsilosis* had relatively high cell surface hydrophobicity. Considering its strong adhesion, robust biofilm formation at 4°C, and high metabolic activity, *C. parapsilosis* is well-equipped to attach to and form biofilms on cold, abiotic surfaces (like stored corneas). We additionally observed that *C. parapsilosis* and *C. tropicalis* hydrophobicity was positively correlated with biofilm biomass, suggesting that cell surface hydrophobicity could serve as another indicator of biofilm-forming capability.

4. The results of corneal secretion smears from patients with postoperative *Candida* keratitis in this study showed that some *Candida* can form biofilms upon infecting donor corneas. In recording colony and spore morphology, we found these characteristics to be associated with biofilm-forming ability, consistent with the findings of Emilia Gómez-Molero et al (Gómez-Molero et al., 2021). Our study showed that *C. parapsilosis* colonies grow more slowly than other *Candida* – non-smooth (rough) colony morphology could only be observed after 48 hours of growth, and became more pronounced by 96 hours, which is different from other *Candida* species. When laboratory conditions limit immediate biofilm testing, one should preliminarily infer biofilm formation ability based on colony morphology and spore microscopic characteristics. For *C. parapsilosis*, the incubation time should be extended to observe colony texture changes. This approach can guide subsequent choice of antifungal agents and dosing.

5. We found that 1, 3-β-D-glucan levels were inversely correlated with biofilm metabolic activity – in other words, faster biofilm formation was associated with weaker antigen expression. Thus, in clinical practice, a low 1, 3-β-D-glucan level (or a negative serum G-test) cannot completely rule out *Candida* infection; clinical judgment should consider risk factors, symptoms, and signs in combination. Early identification of contaminated donor corneas is critical for the prognosis of corneal transplants. Because postoperative ocular infections can be caused by a variety of microbes (not limited to *Candida*), performing metagenomic next-generation sequencing (mNGS) on corneal storage solution prior to transplantation is very important to detect any contamination of the donor cornea. Our study also showed a certain relationship between 1, 3-β-D-glucan release and neutrophil chemotaxis: non-biofilm-forming strains released higher levels of 1, 3-β-D-glucan and attracted more neutrophils than biofilm-forming strains, although the difference was not statistically significant. This suggests that non-biofilm strains, which express higher levels of antigens (mainly 1, 3-β-D-glucan and mannan), can recruit more neutrophils to the infection site. In contrast, biofilm-forming strains attract fewer neutrophils, potentially impairing the host’s ability to fight the infection and leading to prolonged disease.

6. Natamycin and voriconazole are first-line topical antifungals for fungal keratitis. Chlorhexidine is a cationic biguanide disinfectant/antiseptic that exerts broad-spectrum antimicrobial activity mainly by binding to and disrupting fungal cell membranes (Hiom et al., 1996; McDonnell and Russell, 1999; Lima de Sousa et al., 2024). Chlorhexidine can bind to keratin in the corneal epithelium and persist, and studies have confirmed that repeated use has a cumulative effect (Mullany et al., 2006; Abbood et al., 2023). We found that 16 C*. albicans* and 3 C*. tropicalis* isolates had high terbinafine MICs (>16 μg/mL) in planktonic tests, consistent with the findings of Pashootan et al. that this resistance is related to mutations in the squalene epoxidase (SQLE) gene (Pashootan et al., 2022). Research on *Candida* from skin infections also showed that 88.05% of *C. albicans* and 82.14% of *C. tropicalis* isolates are non-wild-type (suggesting potential resistance) to terbinafine (Yang et al., 2022). In the treatment of *Candida* keratitis, oral terbinafine is sometimes used empirically, but our findings indicate that this therapy may be ineffective and could even promote resistance under drug pressure; hence, routine use of terbinafine for ocular *Candida* is not recommended.

Our study showed that *Candida* in biofilm form has substantially reduced susceptibility to antifungal drugs compared to planktonic *Candida*. The MICs of all commonly used antifungal agents reached levels indicative of resistance in the biofilm state. Except for caspofungin, the biofilm MICs of all other antifungal drugs were at resistant or much higher concentrations than their planktonic MICs, consistent with related studies (Ramage et al., 2001; Ruiz de Alegría Puig et al., 2023; Przybek-Skrzypecka et al., 2024). Most antifungal agents target ergosterol in the cell membrane, whereas caspofungin inhibits 1, 3-β-D-glucan synthase, disrupting cell wall synthesis. It also suppresses biofilm formation and reduces the metabolic activity of biofilms (Katragkou et al., 2008). Topical natamycin and other agents may not achieve therapeutic concentrations within a mature biofilm, leading to poor clinical outcomes in post-corneal transplant *Candida* infections and the need for surgical intervention in some patients (Tobudic et al., 2012; Das et al., 2022). Moreover, since *Candida* can form biofilms on corneas during cold storage, the low-temperature environment slows fungal metabolism, and most antifungal drugs are more effective against actively growing cells. Thus, low temperatures induce an increase in biofilm-related drug resistance, rendering many standard antifungals ineffective (Hall and Mah, 2017).

7. Microsatellite genotyping revealed a sporadic distribution of *C. parapsilosis* genotypes, with no closely related cluster of strains identified. This sporadic pattern may be due to patients having undergone ophthalmic surgeries at different hospitals and geographic locations. However, interestingly, all *C. parapsilosis* isolates from 2024 fell into only two genotypes (III and IV), and all were high biofilm-producing strains. This finding suggests that we should monitor the microsatellite genotypes of *C. parapsilosis* isolates in the post-2024 period for any emerging clustering trends, in order to prevent the occurrence of nosocomial outbreaks.

8. We also noted one *C. tropicalis* strain that produced a much greater biofilm at 37°C (far exceeding other *Candida* in biomass) but had poor biofilm formation at 4°C, below most *C. parapsilosis* strains. Although *C. tropicalis* infections should not be underestimated—especially in high-risk settings like ICUs where its incidence exceeds that of *C. parapsilosis* and it shows resistance to some antifungals—this particular observation underscores a key difference: *C. tropicalis* is less adept at forming biofilms at low temperatures compared to *C. parapsilosis*. Thus, while *C. tropicalis* can be highly virulent in certain contexts, in the scenario of cold-stored corneal grafts, *C. parapsilosis* has a distinct advantage and warrants heightened attention (Negri et al., 2012; Wang et al., 2021; Ahmad et al., 2022).

## 5 Conclusion

*C. parapsilosis* is the leading *Candida* species isolated after corneal transplantation, and it exhibits significantly greater adhesion ability and biofilm-forming capacity at 4°C (with higher metabolic activity) than other *Candida* species. This propensity likely contributes to its higher post-transplant infection rate. When a laboratory encounters a *Candida* isolate with rough colony morphology, antifungal susceptibility testing should be performed under biofilm-growing conditions rather than only in the planktonic state. High biofilm-producing strains may yield false-negative results in the G-test (serum 1, 3-β-D-glucan assay), so early identification of biofilm-producing Candida strains is critical. If resources for biofilm testing are limited, clinicians managing post-corneal transplant infections should still account for the possibility of biofilm. When all other antifungal agents prove ineffective, caspofungin should be appropriately considered for treatment.

## Data Availability

The original contributions presented in the study are included in the article/supplementary material. Further inquiries can be directed to the corresponding author.

## References

[B32] Abbood H. M. Hijazi K. Gould I. M. (2023). Chlorhexidine resistance or cross-resistance, that is the question. Antibiotics (Basel). 12, 798. doi: 10.3390/antibiotics12050798 37237701 PMC10215778

[B45] Ahmad S. Kumar S. Rajpal K. Sinha R. Kumar R. Muni S. . (2022). Candidemia among ICU patients: species characterisation, resistance pattern and association with candida score: A prospective study. Cureus 14, e24612. doi: 10.7759/cureus.24612 35651467 PMC9138890

[B8] AsoganM. KimH. Y. KiddS. Alastruey-IzquierdoA. GovenderN. P. DaoA. . (2024). Candida parapsilosis: A systematic review to inform the World Health Organization fungal priority pathogens list. Med. Mycol 62, myad131., PMID: 38935912 10.1093/mmy/myad131PMC11210616

[B4] Bezerra F. M. Rocchetti T. T. Lima S. L. Yu M. C. Z. da Matta D. A. Höfling-Lima A. L. . (2023). Candida species causing fungal keratitis: molecular identification, antifungal susceptibility, biofilm formation, and clinical aspects. Braz. J. Microbiol. 54, 629–636. doi: 10.1007/s42770-023-00964-w 37055625 PMC10235373

[B24] Borges K. R. A. Pimentel I. V. Lucena L. C. L. D. S. Silva M. A. C. N. D. Monteiro S. G. Monteiro C. A. . (2018). Adhesion and biofilm formation of Candida parapsilosis isolated from vaginal secretions to copper intrauterine devices. Rev. Inst Med. Trop. Sao Paulo. 60, e59. doi: 10.1590/s1678-9946201860059 30365642 PMC6199129

[B18] Clinical and Laboratory Standards Institute. (CLSI) (2008). Reference method for broth dilution antifungal susceptibility testing of yeasts. 3rd ed (Wayne: Clinical and Laboratory Standards Institute).

[B17] Clinical and Laboratory Standards Institute. (CLSI) (2012). Reference method for broth dilution antifungal susceptibility testing of yeasts; fourth informational supplement (Wayne: Clinical and Laboratory Standards Institute).

[B26] Cuéllar-Cruz M. Vega-González A. Mendoza-Novelo B. López-Romero E. Ruiz-Baca E. Quintanar-Escorza M. A. . (2012). The effect of biomaterials and antifungals on biofilm formation by Candida species: a review. Eur. J. Clin. Microbiol. Infect. Dis. 31, 2513–2527. doi: 10.1007/s10096-012-1634-6 22581304

[B41] Das S. Chaurasia S. Sharma S. Das S. (2022). Early postoperative infection following lamellar keratoplasty: a review. Br. J. Ophthalmol. 106, 741–754. doi: 10.1136/bjophthalmol-2020-318305 33941590

[B7] Deogaonkar K. Roy A. (2023). Donor related corneal graft infection: a review of literature and preventive strategies. Semin. Ophthalmol. 38, 219–225. doi: 10.1080/08820538.2022.2095873 35787733

[B23] de Souza C. M. Dos Santos M. M. Furlaneto-Maia L. Furlaneto M. C. (2023). Adhesion and biofilm formation by the opportunistic pathogen Candida tropicalis: what do we know? Can. J. Microbiol. 69, 207–218. doi: 10.1139/cjm-2022-0195 36809069

[B21] Dorko E. Baranová Z. Jenca A. Kizek P. Pilipcinec E. Tkáciková L. (2005). Diabetes mellitus and candidiases. Folia Microbiol. (Praha). 50, 255–261. doi: 10.1007/BF02931574 16295665

[B2] Fontana L. Moramarco A. Mandarà E. Russello G. Iovieno A. (2019). Interface infectious keratitis after anterior and posterior lamellar keratoplasty. Clinical features and treatment strategies. A review. Br. J. Ophthalmol. 103, 307–314. doi: 10.1136/bjophthalmol-2018-312938 30355718 PMC6579547

[B15] Giacobbe D. R. Del Bono V. Viscoli C. Mikulska M. (2017). Use of 1, 3-β-D-glucan in invasive fungal diseases in hematology patients. Expert Rev. Anti Infect. Ther. 15, 1101–1112. doi: 10.1080/14787210.2017.1401467 29125373

[B28] Gómez-Molero E. De-la-Pinta I. Fernández-Pereira J. Groß U. Weig M. Quindós G. . (2021). Candida parapsilosis colony morphotype forecasts biofilm formation of clinical isolates. J. Fungi (Basel). 7, 33. doi: 10.3390/jof7010033 33430377 PMC7827155

[B42] Hall C. W. Mah T. F. (2017). Molecular mechanisms of biofilm-based antibiotic resistance and tolerance in pathogenic bacteria. FEMS Microbiol. Rev. 41, 276–301. doi: 10.1093/femsre/fux010 28369412

[B13] HarjaiK. GuptaR. K. SehgalH. (2014). Attenuation of quorum sensing controlled virulence of Pseudomonas aeruginosa by cranberry. Indian J. Med. Res. 139, 446–453., PMID: 24820840 PMC4069740

[B22] Hernández-Pabón J. C. Tabares B. Gil Ó. Lugo-Sánchez C. Santana A. Barón A. . (2024). CandidaNon-albicans and non-auris causing invasive candidiasis in a fourth-level hospital in Colombia: epidemiology, antifungal susceptibility, and genetic diversity. J. Fungi (Basel). 10, 326. doi: 10.3390/jof10050326 38786681 PMC11122357

[B29] HiomS. J. FurrJ. R. RussellA. D. HannA. C. (1996). The possible role of yeast cell walls in modifying cellular response to chlorhexidine diacetate. Cytobios 86, 123–135., PMID: 9022261

[B16] Justus C. R. Marie M. A. Sanderlin E. J. Yang L. V. (2023). Transwell *in vitro* cell migration and invasion assays. Methods Mol. Biol. 2644, 349–359. 10.1007/978-1-0716-3052-5_22 PMC10335869 37142933

[B39] Katragkou A. Chatzimoschou A. Simitsopoulou M. Dalakiouridou M. Diza-Mataftsi E. Tsantali C. . (2008). Differential activities of newer antifungal agents against Candida albicans and Candida parapsilosis biofilms. Antimicrob. Agents Chemother. 52, 357–360. doi: 10.1128/AAC.00856-07 17938192 PMC2223899

[B6] Lau N. Hajjar Sesé A. Augustin V. A. Kuit G. Wilkins M. R. Tourtas T. . (2019). Fungal infection after endothelial keratoplasty: association with hypothermic corneal storage. Br. J. Ophthalmol. 103, 1487–1490. doi: 10.1136/bjophthalmol-2018-312709 30563913

[B31] Lima de Sousa T. Dourado D. Rodrigues J. S. de Souza Rebouças J. Montes M. A. J. R. Formiga F. R. (2024). Treatment of periodontal disease: does drug delivery matter? Front. Bioeng Biotechnol. 12, 1427758. doi: 10.3389/fbioe.2024.1427758 39081330 PMC11286396

[B3] Masoumi A. Soleimani M. Azizkhani M. Izadi A. Cheraqpour K. Tabatabaei S. A. . (2024). Clinical features, risk factors, and management of candida keratitis. Ocul Immunol. Inflamm. 32, 1169–1174. doi: 10.1080/09273948.2023.2203752 37141453

[B30] McDonnell G. Russell A. D. (1999). Antiseptics and disinfectants: activity, action, and resistance [published correction appears in Clin Microbiol Rev 2001 Jan;14(1):227. Clin. Microbiol. Rev. 12, 147–179. doi: 10.1128/CMR.12.1.147 9880479 PMC88911

[B33] Mullany L. C. Darmstadt G. L. Khatry S. K. Katz J. LeClerq S. C. Shrestha S. . (2006). Topical applications of chlorhexidine to the umbilical cord for prevention of omphalitis and neonatal mortality in southern Nepal: a community-based, cluster-randomised trial. Lancet 367, 910–918. doi: 10.1016/S0140-6736(06)68381-5 16546539 PMC2367116

[B43] Negri M. Silva S. Henriques M. Oliveira R. (2012). Insights into Candida tropicalis nosocomial infections and virulence factors. Eur. J. Clin. Microbiol. Infect. Dis. 31, 1399–1412. doi: 10.1007/s10096-011-1455-z 22037823

[B34] Pashootan N. Shams-Ghahfarokhi M. Chaichi Nusrati A. Salehi Z. Asmar M. Razzaghi-Abyaneh M. (2022). Phylogeny, antifungal susceptibility, and point mutations of SQLE gene in major pathogenic dermatophytes isolated from clinical dermatophytosis. Front. Cell Infect. Microbiol. 12, 851769. doi: 10.3389/fcimb.2022.851769 35372131 PMC8972121

[B12] PierceC. G. UppuluriP. TristanA. R. WormleyF. L. MowatE. RamageG. . (2008). A simple and reproducible 96-well plate-based method for the formation of fungal biofilms and its application to antifungal susceptibility testing. Nat. Protoc. 3, 1494–1500. doi: 10.1038/nprot.2008.14118772877 PMC2741160

[B38] Przybek-Skrzypecka J. Samelska K. Ordon A. J. Skrzypecki J. Izdebska J. Kołątaj M. . (2024). Post-keratoplasty microbial keratitis in the era of lamellar transplants-A comprehensive review. J. Clin. Med. 13, 2326. doi: 10.3390/jcm13082326 38673599 PMC11051457

[B36] Ramage G. Vande Walle K. Wickes B. L. López-Ribot J. L. (2001). Standardized method for *in vitro* antifungal susceptibility testing of Candida albicans biofilms. Antimicrob. Agents Chemother. 45, 2475–2479. doi: 10.1128/AAC.45.9.2475-2479.2001 11502517 PMC90680

[B10] Rodríguez-Temporal D. Díez R. Díaz-Navarro M. Escribano P. Guinea J. Muñoz P. . (2023). Determination of the ability of matrix-assisted laser desorption ionization time-of-flight mass spectrometry to identify high-biofilm-producing strains. Front. Microbiol. 13, 1104405. doi: 10.3389/fmicb.2022.1104405 36704568 PMC9871577

[B37] Ruiz de Alegría PuigC. García MerinoM. D. S. De Malet Pintos-FonsecaA. Agüero BalbínJ. (2023). Characterization, antifungal susceptibility and virulence of Candida parapsilosis complex isolates in a tertiary hospital in Cantabria, Northern Spain. Enferm Infecc Microbiol. Clin. (Engl Ed). 41, 99–102., PMID: 36759059 10.1016/j.eimce.2022.11.016

[B19] Sabino R. Sampaio P. Rosado L. Stevens D. A. Clemons K. V. Pais C. (2010). New polymorphic microsatellite markers able to distinguish among Candida parapsilosis sensu stricto isolates. J. Clin. Microbiol. 48, 1677–1682. doi: 10.1128/JCM.02151-09 20220157 PMC2863883

[B27] Silva S. Henriques M. Oliveira R. Williams D. Azeredo J. (2010). *In vitro* biofilm activity of non-Candida albicans Candida species. Curr. Microbiol. 61, 534–540. doi: 10.1007/s00284-010-9649-7 20401483

[B25] Silva S. Negri M. Henriques M. Oliveira R. Williams D. W. Azeredo J. (2011). Adherence and biofilm formation of non-Candida albicans Candida species. Trends Microbiol. 19, 241–247. doi: 10.1016/j.tim.2011.02.003 21411325

[B14] Silva-Dias A. Miranda I. M. Branco J. Monteiro-Soares M. Pina-Vaz C. Rodrigues A. G. (2015). Adhesion, biofilm formation, cell surface hydrophobicity, and antifungal planktonic susceptibility: relationship among Candida spp. Front. Microbiol. 6, 205. doi: 10.3389/fmicb.2015.00205 25814989 PMC4357307

[B1] Song A. Deshmukh R. Lin H. Ang M. Mehta J. S. Chodosh J. . (2021). Post-keratoplasty infectious keratitis: epidemiology, risk factors, management, and outcomes. Front. Med. (Lausanne). 8, 707242. doi: 10.3389/fmed.2021.707242 34307431 PMC8292647

[B11] Tavanti A. Hensgens L. A. Mogavero S. Majoros L. Senesi S. Campa M. (2010). Genotypic and phenotypic properties of Candida parapsilosis sensu strictu strains isolated from different geographic regions and body sites. BMC Microbiol. 10, 203. doi: 10.1186/1471-2180-10-203 20667137 PMC2919483

[B5] Thareja T. Kowalski R. Kamyar R. Dhaliwal D. Jeng B. H. Tu E. . (2020). Fungal infection after keratoplasty and the role of antifungal supplementation to storage solution: a review. Br. J. Ophthalmol. 104, 1036. doi: 10.1136/bjophthalmol-2019-314664 31796428

[B40] Tobudic S. Kratzer C. Lassnigg A. Presterl E. (2012). Antifungal susceptibility of Candida albicans in biofilms. Mycoses 55, 199–204. doi: 10.1111/j.1439-0507.2011.02076.x 21793943

[B44] Wang B. He X. Lu F. Li Y. Wang Y. Zhang M. . (2021). Candida isolates from blood and other normally sterile foci from ICU patients: determination of epidemiology, antifungal susceptibility profile and evaluation of associated risk factors. Front. Public Health 9, 779590. doi: 10.3389/fpubh.2021.779590 34858938 PMC8632017

[B9] Xie T. A. Liu Y. L. Liang C. Huang Y. Y. Li J. W. Li Z. W. . (2019). Accuracy of matrix-assisted LASER desorption ionization-time of flight mass spectrometry for identification of Candida. Biosci. Rep. 39, BSR20190859. doi: 10.1042/BSR20190859 31537628 PMC6822510

[B35] Yang Z. Zhang F. Li D. Wang S. Pang Z. Chen L. . (2022). Correlation between drug resistance and virulence of candida isolates from patients with candidiasis. Infect. Drug Resist. 15, 7459–7473. doi: 10.2147/IDR.S387675 36544991 PMC9762413

